# An analysis of the possibilities and challenges of long stay tourism in Thailand

**DOI:** 10.12688/f1000research.128437.2

**Published:** 2023-11-06

**Authors:** Warach Madhyamapurush

**Affiliations:** 1 Tourism and Hotel Management, School of Business and Communication Art, University of Phayao, Phayao, Thailand

**Keywords:** lon-stay tourism, Thailand, possibilities and challenges, current status, tourist business, tourist attractions

## Abstract

The tourism sector is significant in emerging nations like Thailand. The cost of lodging is a significant component of practically every trip, thus it is important to consider accommodation development while trying to draw in visitors from other nations. The long-stay tourism industry is crucial since longer visitor stays result in more revenue. Following this, other research on long-term lodging of all forms has been conducted, with an emphasis on both the tourist and real estate sectors. The best tourist option in Thailand is long-term travel. As evidenced by the American, European, and Japanese visitors, the target market is tourists from nations with high costs of living, frigid climates, and aging populations. Therefore, it is anticipated that the tourist demographic will change in future, leading to the emergence of the retirement home niche market as a part of long-stay tourism. The characteristics of long-stay tourism in Thailand are examined in this paper, and we assess the theoretical and conceptual framework as an analysis of Thailand's tourism. Examining the current situation of the Thai long-stay tourist business is the initial and main objective of this study. There is currently no perfect answer, but various alternatives from comparable markets in representative nations have been used as examples to subsequently create tourism accommodation in Thailand for long-stay tourism.

## 1. Introduction

**Figure 1.**
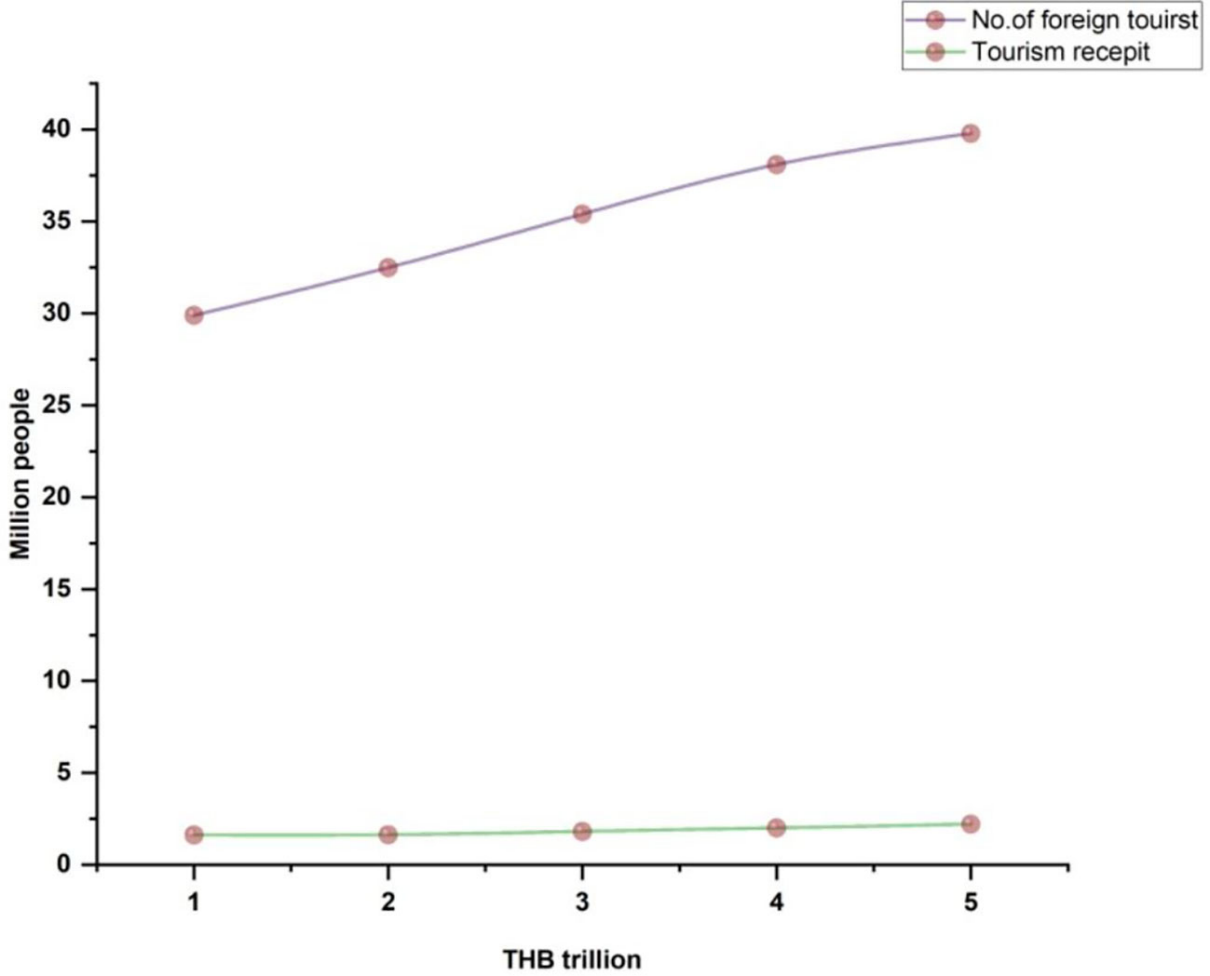
Thailand international tourist arrival.

One type of tourism that has been popular for a long time is long-stay travel. Spain is without a doubt the most popular travel destination, but due to the increased globalization of the travel industry, growing regions like Southeast Asia are already showing remarkable market shares for long-stay travel. ^1^ Apart from leisure, long-term travel is heavily influenced by the concept of a “second home,” which is common parlance among visitors with disposable income. As a result, the word “retirement home” is also closely associated because of the global socio-demographic trend, which foresees a significant shift in the proportion of the population that is older in several nations, including Japan and Scandinavian countries. ^2^ The Japanese are a prominent foreign ethnic minority in Thailand and have a long-term presence there for a variety of reasons, including employment, travel, education, and retirement. ^1^ Figure 1 indicates Thailand's international tourist arrivals.

Due to the concern for an aging population, the retiree group is significant. Additionally, the Japanese government and the Japan & Thailand International Relations Organization (JTIRO) both strongly support this group. Japanese visitors and senior citizens have stayed in a variety of lodging options, including hotels, apartments for rent, and condominiums. In contrast to the Japanese, Europeans do not provide any kind of assistance to their elderly tourists. However, due to their potential and active efforts in reserving long-term lodgings in Thailand, European visitors, particularly those from Scandinavia, are a significant segment. Although there is no official database of long-term lodgings, many coastal tourist destinations have evidence of their presence along the shore. The Thai government investigated and conducted several efforts to encourage long-stay tourism, particularly the retirement segment, after realizing the importance of the sector. The 2004 campaign was unsuccessful thus far for a variety of reasons, and it is necessary to revise it to draw tourists and compete with other nations. In this study, the terms “long-stay accommodation” and “long-stay tourism” will be used synonymously for convenience. ^2^ After the global economic crisis of 2008, it became abundantly clear that the tourist industry was a mainstay of the Thai economy. When the crisis first started, it didn't have much of an impact on Thailand. This was due largely to the fact that the tourism industry was responsible for a sizable portion of the country's GDP, and so provided a large number of people with employment opportunities. ^3^^,^^4^ Thailand is a nation whose economy heavily relies on the travel and tourism sector. Thailand's natural beauty in Southeast Asia has earned it the nickname “Land of Smiles.” Many individuals in Thailand depend on the tourism sector to provide a living. Thailand, which is ranked as the ninth nation with the most money generated by tourism, has received THB 2000 yearly since 2014, according to the Tourism Authority of Thailand (TAT). Thailand also drew 32.6 million tourists globally in 2016, placing ninth overall and second in Asia. Thailand's foreign visitors have greatly expanded, and because of this quick expansion, the country placed fourth globally in terms of incoming tourists in 2015. ^5^ Thailand's capital, Bangkok, continued to rank second among the most popular cities for international travelers. The fourth quarter of 2016 saw challenges for Thailand's tourism industry as a result of the Thai government's efforts to combat illegal immigration and impose various entertainment-related regulations. The growth of the tourist sector, however, remained steady. Additionally, the foreign tourist receipts for Thailand's tourism business were $49.9 billion in 2016 and $44.9 billion in 2015. In 2016, these earnings made up 12.3% of Thailand's GDP. This significant portion of the income from foreign visitors, primarily from China and Europe, is broken down as follows: Europe (THB 460 billion), China (THB 439 billion), America (THB 102 billion), CLMV2 (THB 97 billion), the Middle East (THB 5 billion), and other regions (THB 489 billion). ^3^ The original and primary goal of this review is to examine the state of the Thai long-stay tourism market today. The link between the growth of long-stay accommodation and tourist trends will be studied to comprehend the market. Additionally, all appropriate tourist options will be illustrated to encourage long-stay tourism in the market.

The remaining section of the article is divided into five sections: Section 2 – Methods, Section 3 – Results, Section 4 – Discussion, and Section 5 –Conclusions.

## 2. Methods

**Figure 2.**
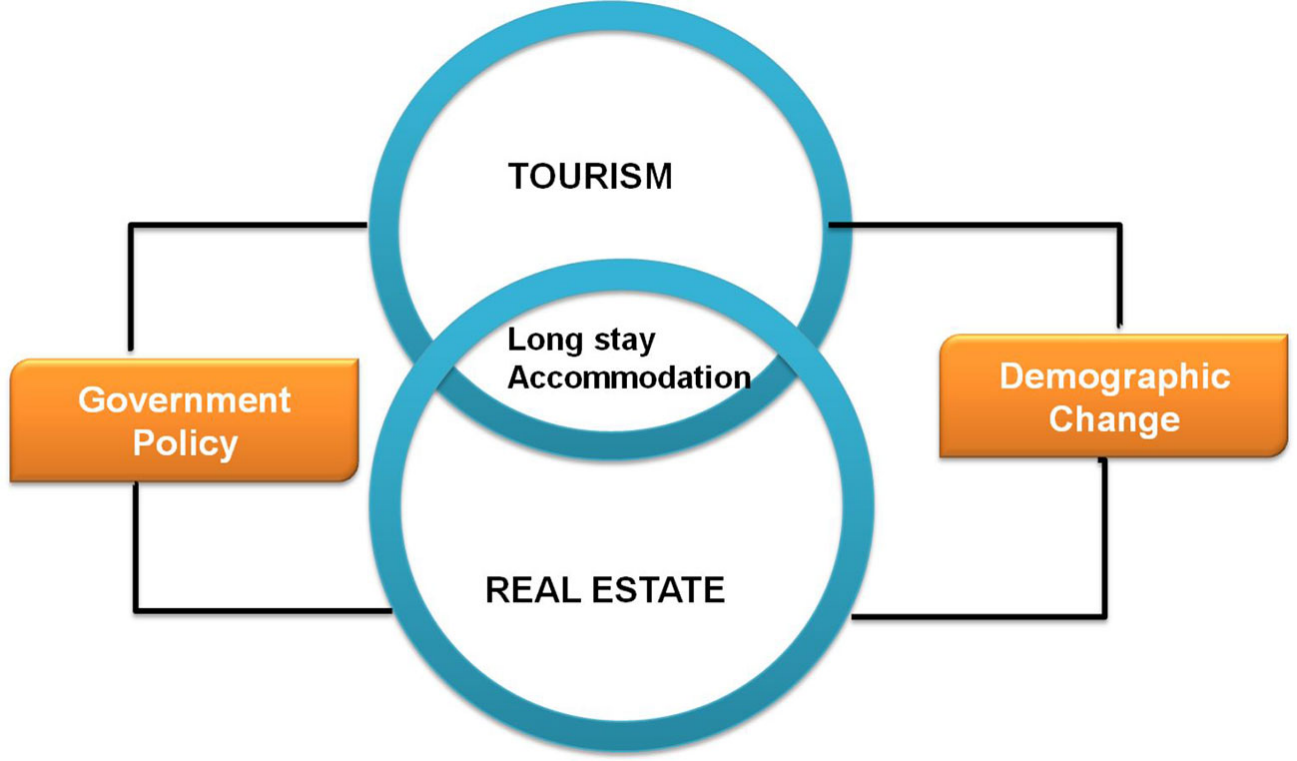
Conceptual framework.

This section introduces prior research pertinent to the goals and objectives of the thesis. This section presents four components. First, the purpose of tourism reveals the nature and fundamental components of tourism. ^6^ The role of demographic shift is then illustrated to highlight the anticipated future trend in tourism. Additionally, as lodging is a fundamental component of tourism, long-term lodging ownership is demonstrated. Domestic and foreign travel were both prohibited during the lockdown and preventive measures' implementation period, severely reducing earnings from tourism-related business. This revenue accounted for around 12% of Thailand's GDP and 50% of Thailand's export GDP, the latter being further impacted by the coronavirus disease (COVID-19) induced global slump. ^7^ Finally, this section explains why government policy must regulate the real estate market. The conceptual foundation for the study is briefly illustrated as seen in Figure 2.

### 2.1 The purpose of tourism and long-term accommodation

**Figure 3.**
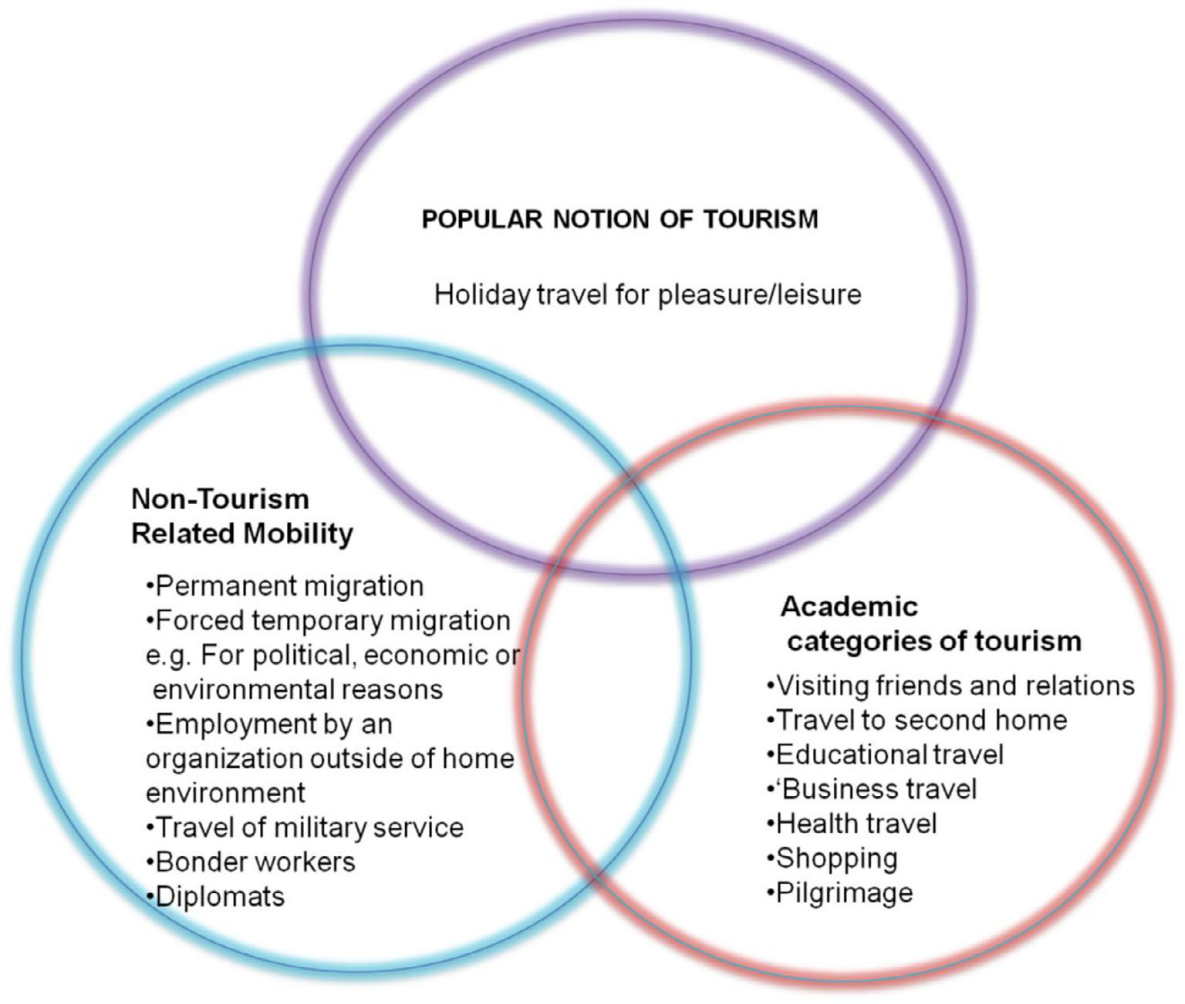
Academic and popular perceptions of tourism.

The 1992 Earth Summit in Brazil, which gave rise to the present worldwide emphasis on sustainable development, has acted as a driving force for the growth of tourism in three important areas. Protecting the natural resources and the environment is essential, followed by the need to continuously research the tourist industry, and finally, the need to develop human resources. All of these important factors have driven alternative tourism development to meet not only the recognized demands, but also to replace current tourism growth. Long-stay tourism is seen as an example of alternative tourism since it emphasizes language acquisition and intercultural interactions between visitors, who are viewed as guests, and locals, who are viewed as hosts. Because lodging is one of the three fundamental components of tourism—the other two being transportation and service—the tourist sector is related to real estate acquisition. The shared traits of the notion of a second home, which is based on two primary components, may explain long-stay tourism. Tourists may stay in privately operated, leased, or even free accommodations in this category. Second, they regularly go back to the same vacation spot. Their returns show that they are extremely knowledgeable of, devoted to, and appreciative of the location. To attract and keep visitors, tourism service information must be current, accurate, and easily accessible via a variety of digital mediums. ^8^ As illustrated in Figure 3, Hall has offered three categories of tourism. ^8^ Every visit, however, has the potential to serve many purposes and to straddle multiple categories, as in the case of a tourist who spends their winter vacation at their second home in a warmer country due to forced temporary migration (factors not related to tourism, academic and job opportunities). ^9^ Accommodations that enable Muslim visitors to do the five necessary morning prayers, that must be done at specified times of day (before dawn, in the afternoon, in the afternoon, at sunset, and at night), are a much-appreciated addition to urban landscapes. With many prayer times spread out throughout the day, it's helpful for Muslim travelers to have a clean, well-maintained place to worship before continuing with their day. ^10^

One month or more is regarded as a period of lengthy residence. ^11^ Due to this circumstance, senior or pensioner travelers have emerged as one of the major target demographics for long-term travel. They are finished with their family caring duties and have no further work obligations. ^12^ Additionally, they get regular income from pension funds granted by their government, including elderly visitors from Japan, the United States (US), and Europe. In a nutshell, the following are some elements that favor its development:

• A general increase in household income
• Longer vacations and more free time
• Liberalization of financial flows
• More affordable and effective transportation easing of border restrictions and facilitation of international travel.

The European Union is the second-largest market, with US visitors accounting for the largest portion of second-home tourism. ^13^ There is a rise in demand for such residences in southern European nations, and northern Europeans frequently acquire them. ^14^ One of the frequent customs in the area, like Scandinavian nations, is the ownership of second homes that are essentially household spaces. However, due to frequent travel in some nations, buying second houses or vacation homes overseas has grown in popularity. Spain is a favorite destination for tourists planning extended stays because of its proximity to the Mediterranean Sea, its cultural diversity, and its continued European character. ^15^ At the same time, South American and Central American vacation spots like Costa Rica, Panama, and Brazil are prioritized by travelers from the US. More marketplaces exist now outside of western nations. The primary driver of the initial demand was the need for improved climatic conditions. Transferring to Asian nations, particularly Southeast Asian nations like Malaysia, the Philippines, Singapore, and Thailand, is a significant development area for this industry. ^16^

The second-home vacation sector is dominated by travelers from the US, with visitors from the European Union making up the second-smallest marketplace. The demand certainly rises in the nations of southern Europe, while northern Europeans are the most common buyers of such properties (Pedro, 2006). One of the things that people in the region like Scandinavian nations have in common is the culture of having a second house that is mostly in the domestic area. On the other hand, because of the frequent travel required in some nations, purchasing a vacation or second home in other countries has become an increasingly common practice. Spain is frequently cited as one of the best places in the world for extended vacations due to the country's proximity to the Mediterranean Sea, its diversity of cultural traditions, and its preservation of a European ambiance. At the same time, travelers from the US tend to prioritize locations in South and Central America, such as Brazil, Costa Rica, and Panama. In today's world, there are a growing number of marketplaces that are located outside of western nations. The primary impetus behind initial demand was consumers' desire to experience more favorable weather conditions. ^14^ The relocation of operations to Asian nations, particularly those in Southeast Asia like Malaysia, the Philippines, Singapore, and Thailand, is one of the most significant drivers of growth in this industry.

### 2.2 The impact of demographic changes and long-stay accommodation

After the homecoming of American soldiers from World War II, the term “Baby Boomer” was first used to describe Americans born between 1946 and 1964. Despite efforts to contain the COVID-19 epidemic by limits on international travel, these restrictions cannot be seen as viable long-term answers to the situation. When people are unable to freely explore a country, it can have devastating effects on the economy, including a deepening recession and a drop in tourism revenue in Thailand. ^17^ The U.S. birth rate skyrocketed during that period, and they today make up an age range between 42 and 65 years. The word has been used widely since then to refer to those who were born during that period worldwide, not only in the US. While they are currently experiencing relatively slow demographic development, Europe and Japan will certainly experience some population loss in the first part of the twenty-first century. According to the United Nations, an estimated world population of up to 2300 was estimated, despite not being carried out by the primary area or specific nations. Except for Northern America, almost all areas predicted decreased growth rates. It is believed that the average lifespan will continue to increase indefinitely. Population aging emerges as a key demographic trait outside of the demographic window. Thus, it could cause international migration. While some seniors look for retirement destinations to maintain their pensions, the influx of foreign workforces will address the population and new generational decline. ^18^ Analyzing the recent growth of Thailand's golf tourism sector reveals a market shift in the direction of focusing on improving the tourist industry's management mechanisms and expanding its use of environmentally friendly practices. Golf tourism in Thailand has been around longer than in China, and its development method and talents are more refined. ^14^ There are several ways in which tourism affects the economies of host countries, including the creation of jobs, the building of infrastructure, the expansion of the tourism-related value chain, and other societal and economic ripples across the local population. ^19^

Based on research conducted by the Population Division, DESA, and the United Nations. ^17^ The Japanese are a clear example of the rising elderly population. The government has a program to relocate most elderly pensioners to locations that are suited for them to live out their remaining years with a fixed amount of pension. They willfully purchase products and services that support independent living. Their worries about aging and death have sparked the creation of novel goods and services. The creation of specialized goods and services in the fields of health and medical care, home care, real estate, construction, financial services, education and learning, cuisine, cosmetics, travel, and entertainment are all part of the silver market. Most of the real estate and personal financial assets are owned by seniors, and when these assets are passed down through inheritance, there will be a greater need for new financial management services that include more investment than saving and making use of personal financial assets. Thailand, Malaysia, and the Philippines are three popular travel destinations. Given that Japan is a sizable trading partner and a popular tourist destination, it seems to sense that some of them would choose Thailand as their retirement residence. Currently, a bilateral company oversees other Japanese retirement complexes, and some Japanese families also maintain their private residences for both short-term accommodation and retired living.

### 2.3 Ownership of long-stay accommodation

**Figure 4.**
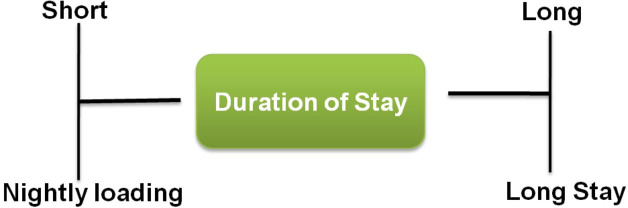
Analogy of a timeline of the duration of stay.

The real estate sector, like many other economic sectors, is an essential component and the foundation of the tourism business. In terms of use patterns, the lines between real estate and tourism are becoming blurred. For instance, hotels operate as both long-term dwellings and workplaces for a mobile workforce of businesspeople, and resort areas provide both residential and recreational facilities to a frequently global populace. The hotel sector is a major topic of empirical examination of the globalization of tourism, particularly the real estate implications. Figure 4 represents the analogy of a timeline of the duration of stay.

## 3. Results

**Figure 5.**
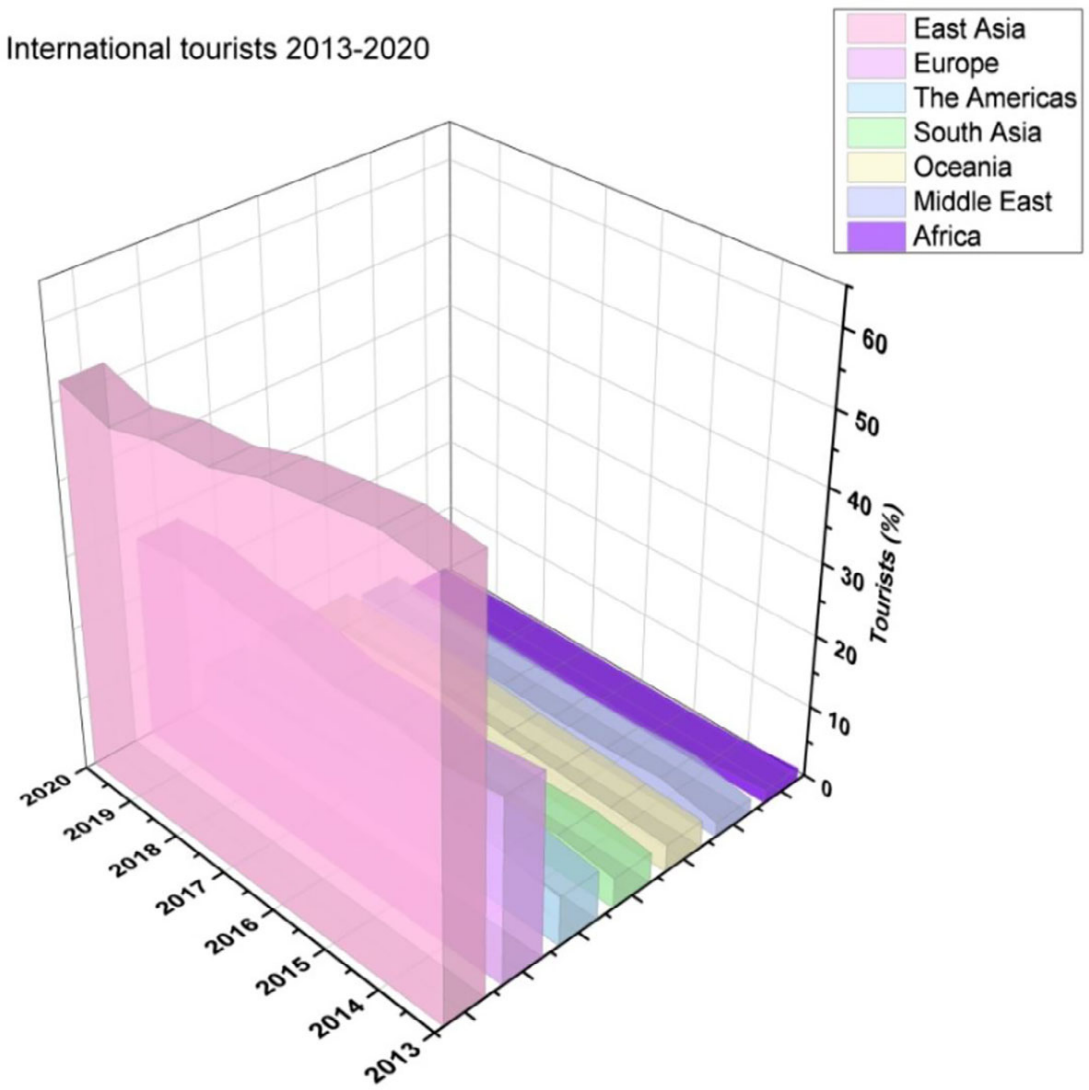
International tourists from 2013 until 2020.

As mentioned, the service sector, which is a key engine for the area, includes both the construction industry and tourism. ^20^ Thailand's tourist prospects are shared by those of its neighbors. Despite the changes throughout this time, the TAT reports that a rise in the number of foreign visitors occurred practically year between 2010 and 2017. Figure 5 illustrates the international tourists from 2010 to 2017.

### 3.1 Thai long-stay market for real estate

Four distinct market categories have been singled out by the TAT as possible long-stay travelers visiting Thailand. The four groups of long-term visitors are as follows:

• Retirees from different nations, with a focus on the Japanese market; the European market, including those from Britain, Germany, the Netherlands, and Scandinavia; the Chinese industry abroad, as well as from other countries. ^21^
• Snowbirds are visitors from chilly nations who will arrive in course of the wintertime. These folks have previously traveled to Thailand to spend two to three weeks taking part in beach activities, experiencing mountain and rural hill tribal life, and engaging in thrilling and daring events. Some people are choosing Thailand as a launching point to visit neighboring nations like Myanmar, Laos, and Cambodia (Indochina). ^22^
• Included in this category are international students and trainees. Numerous education systems choose to establish their affiliation or subsidiaries there to train foreign students in a range of areas due to Thailand's financial advantages. Even though students may not be big spenders, their participation will increase national income.
• Those who go to sports training camps belonging to the fourth category. To conduct their programs at various locations around the nation, several sports training associations have traveled to Thailand with their coaches and players.

As a result, there are four kinds of long-stay accommodation in Thailand:

• A resort or hotel
• Apartments homes and serviced residences
• a targeted health promotion effort
• Accommodations for a certain Group

### 3.2 Market analysis

*3.2.1 Market overview for retail*

**Figure 6.**
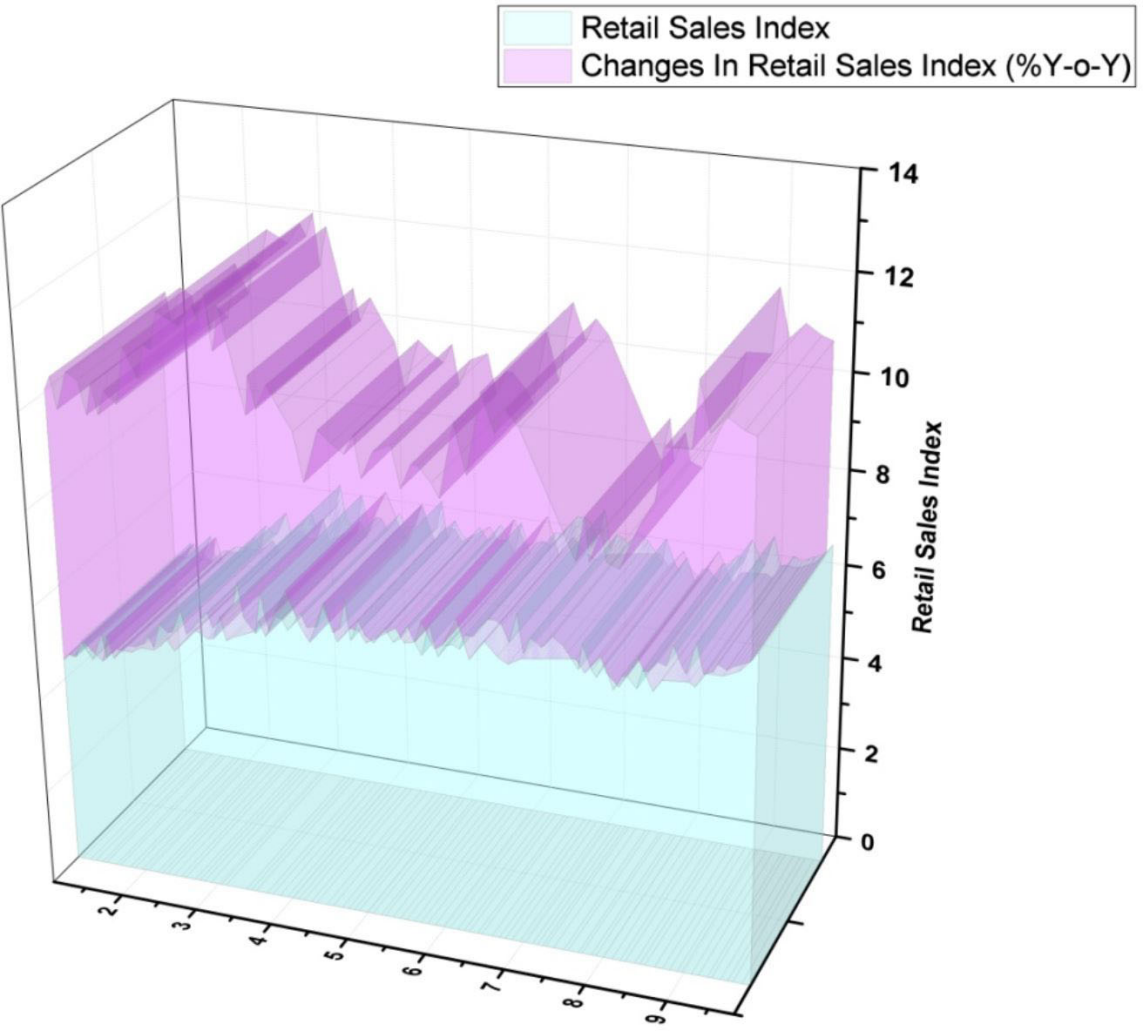
Thailand retail sales index.

**Figure 7.**
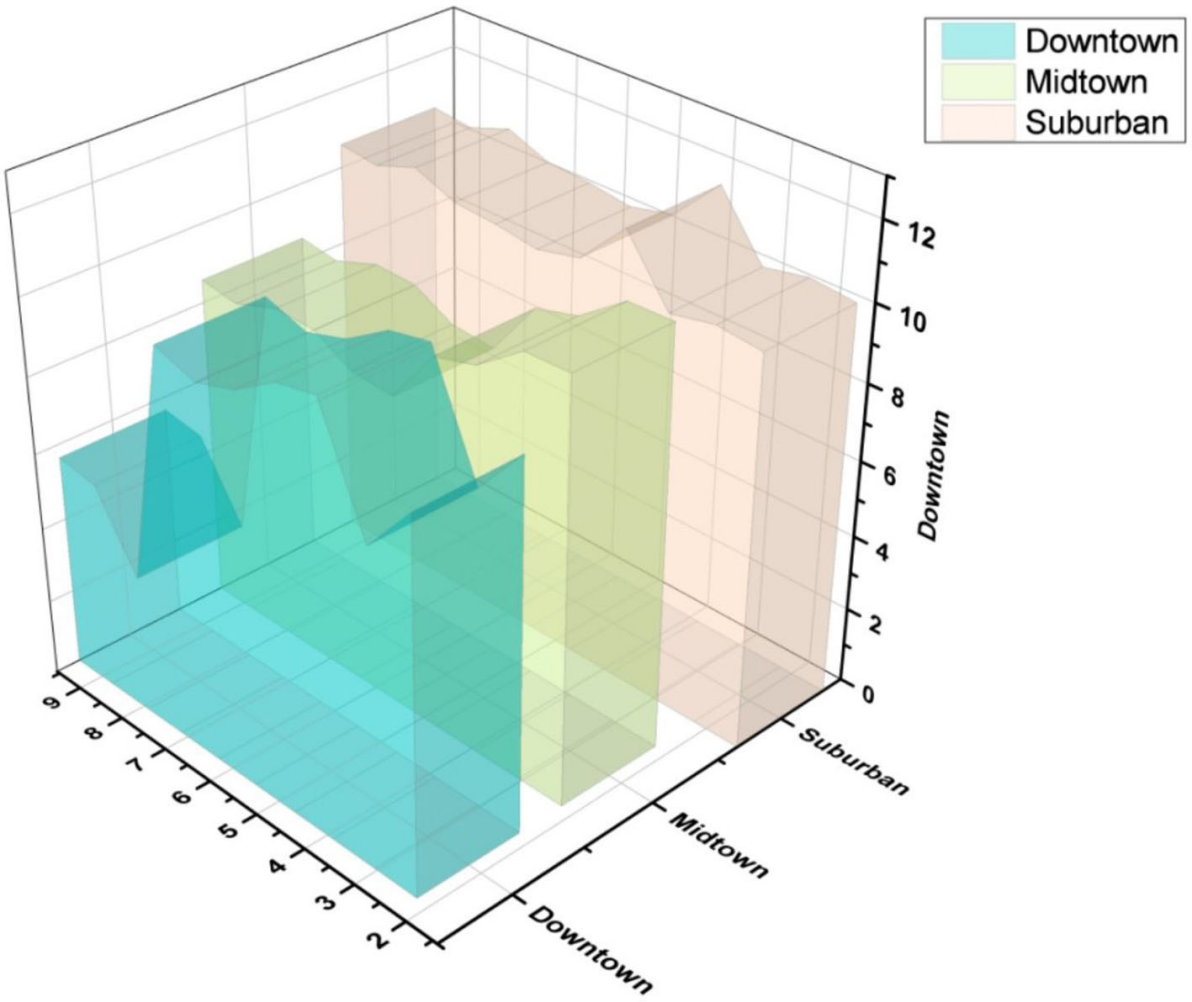
Demand for occupancy by region.

The retail sector in Thailand may be divided into seven major groups based on size, characteristics, products sold, and price, according to a Colliers Thailand analysis. ^23^ After the fourth quarter of 2004, the Bangkok retail market declined. It then rose in 2007, before sharply declining in response to the most recent financial crisis and the protests in 2008–2010. Despite reduced interest rates, the household continued to save due to anxiety over expenditures. Due to planned enhancement, the new future supplies are anticipated to be more fiercely competitive. ^24^ The outdated malls are undergoing renovations or refurbishing to maintain their client base, which would likely cause a decline in occupancy throughout these changes. Figure 6 depicts the Thailand retail sales index. Bangkok's overall retail supply increased to 5.46 million m ^2^, up 0.7% quarter over quarter (Q-o-Q) and 1.5% year over year (Y-o-Y). ^25^ Nevertheless, a rise in customer confidence was accompanied by higher rental rates of 92% (4.97 million m ^2^) accounting for 0.3% Q-o-Q but was still a drop of 1.5 % Y-o-Y. The following figures demonstrate as previously stated. Figure 7 indicates the demand for occupancy by region.

*3.2.2 Demand analysis*

Material obtained from the latest statistics is updated using estimates, while forecasts try to gauge potential changes. Increases in population and employment, facility relocations, speculative demand, and other factors are a few examples of demand drivers. A demand estimate for long-term housing is correlated with the number of visitors and retirees, just like in the tourism sector. A forecast will produce a projection using the number of tourists and demographic changes. The number of long-stay visitors is tracked by the arrival and departure stamps, but it is unclear how many long-stay lodgings there are because some properties are unwittingly acquired as private assets. Real estate items that developers had initially released for their locals but later became well-known in the tourism sector started to be purchased by tourists. ^26^ International visitors and investors have undoubtedly been provided with local coastline and island tourism projects during the previous ten years. Since Thai owners are included in the total, it does not, however, provide an accurate indication of the level of demand from international buyers.

*3.2.3 Supply analysis*

According to TAT's four categories of long-term accommodations, most real estate items for sale fall into the “hotel and resort” and “condominium and service apartment” categories. ^27^

3.2.3.1 Resort and hotels

In conclusion, the amount of tourist lodging in this category is based on the “number of lodging places for tourists,” which adds up to four different types of lodging: hotel, resort, guesthouse, and bungalow. The following are succinct summaries of these four types:

➢ A hotel is a type of housing that is especially designed, separated into rooms, equipped with useful services for tourists, and charges per room.
➢ A resort is a location where guests may rent individual rooms or entire units while enjoying the natural surroundings.
➢ A guest house is a home that has been altered or added to, with split rooms used for accommodation and a rent collection system.
➢ A bungalow is a type of housing where guests can stay in groups, including businesses and tourists, and where rent is charged.

**Figure 8.**
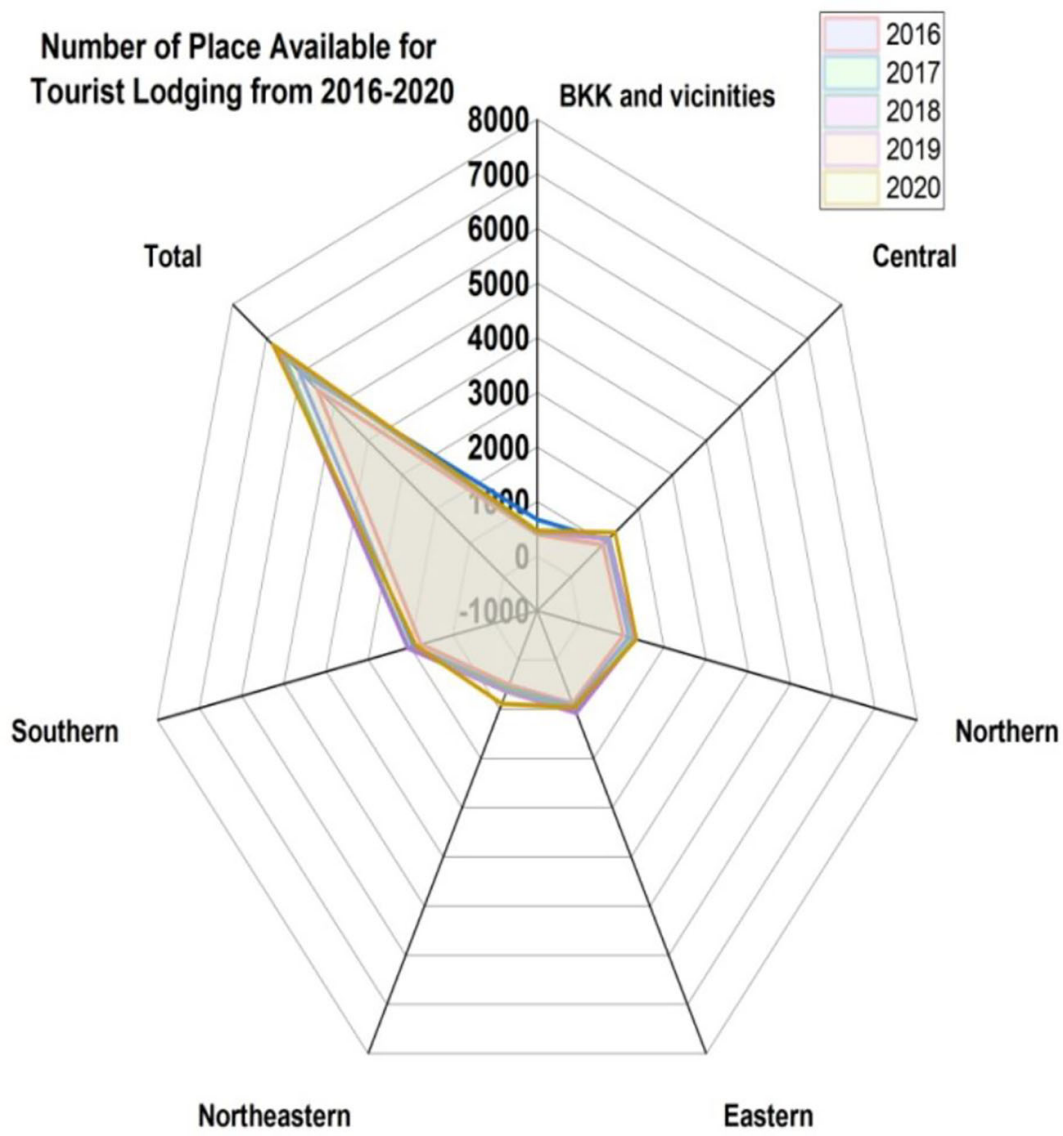
Number of places available for tourist lodging from 2016 to 2020.

**Figure 9.**
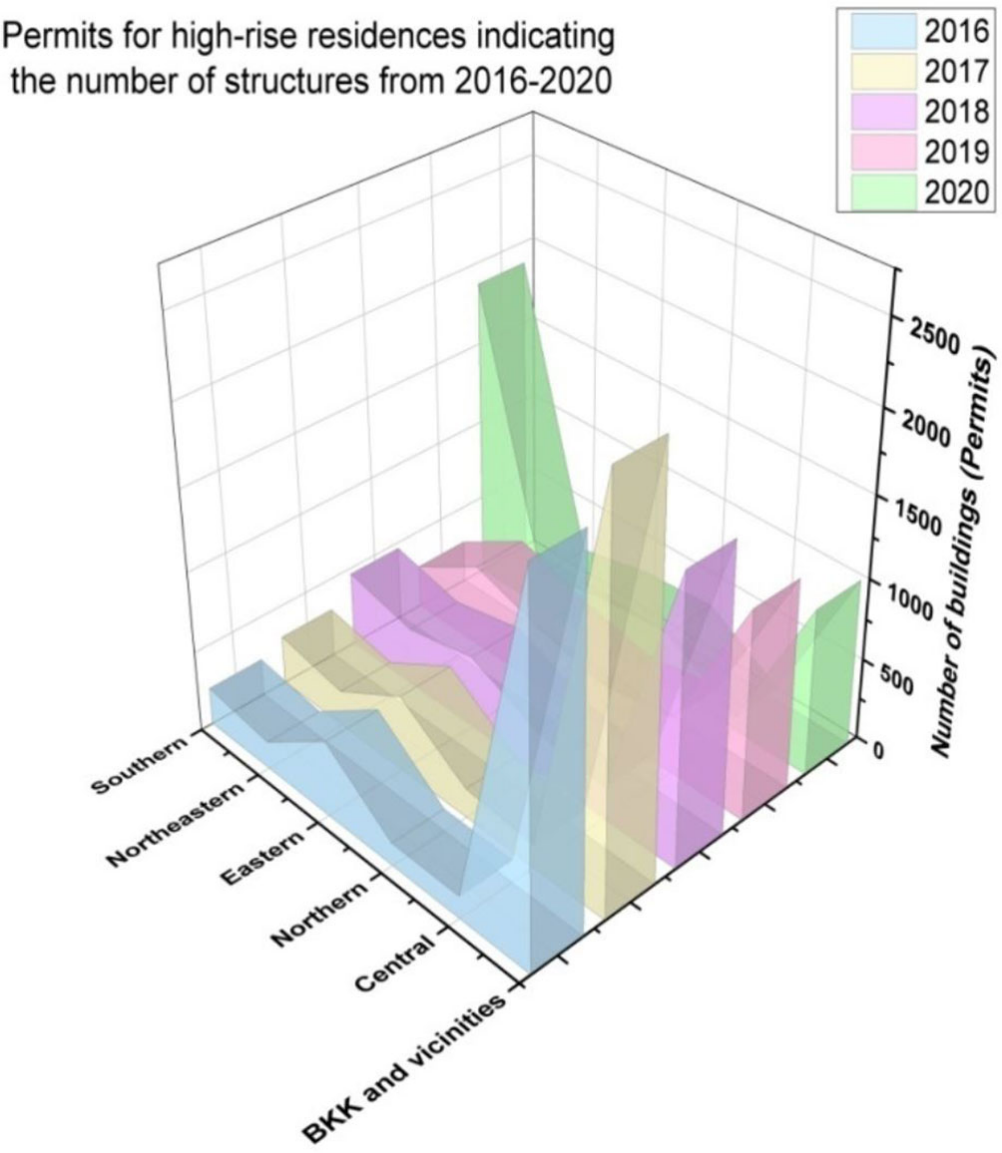
Permits for high-rise residences, indicating the number of structures from 2016 to 2020.

Figure 8 represents the number of places available for tourist lodging from 2016 to 2020. We assess the BKK (BKK is IATA airport code for **Bangkok)** and vicinities, central, northern, eastern, northeastern, and southern. Figure 9 indicates the permits for high-rise residences, indicating the number of structures from 2016 to 2020. ^28^

## 4. Discussion

Ultimately, the study has demonstrated the core tourism potential of Thailand, including its tourist destinations, infrastructure, and lodging. Demand and supply for long-stay accommodations are anticipated to follow the tourist sector as an extraction from long-stay travel, as it formerly did with Spain's Mediterranean coast. Demographic factors are more likely to cause international migration. Tourists, especially frequent tourists, were accustomed to stealing foreign property. However, because of the lodging records' hazy ownership, the study was unable to determine how many accommodations were inhabited by foreign long-stay visitors. Due to ownership restrictions, the names of Thai spouses or Thai companies are frequently used as the owners of properties, which results in this issue. In our case study on Scandinavian visitors, the figure reveals that the number of visitors has been rising in recent years despite minor volatility over the previous 10 years. Interviews with current developers and examples of specific projects have demonstrated the ongoing business. Although Thailand has been a popular destination, it cannot claim to be the most prosperous country. ^29^ The nation has recently been dealing with significant challenges including political unrest, economic fluctuations in the Thai baht, and rising oil prices. ^30^ To further understand how people on minimum wage make sense of Simplified Employee Pension (SEP) and incorporate it into their conceptions of a livable wage, a qualitative study was conducted. Twelve workers at a riverside resort in Thailand were asked to participate in the study because they were representative of employees who had internalized SEP in the workplace. ^31^ These challenges have the consequence that political instability prevents the implementation of consistent policies, such as those that promote long-term travel or even concentrate on senior housing. Additionally, political uncertainty reduces the trust of foreign investors, which reduces investment in other industries. Additionally, currency fluctuations will have an impact on travelers’ decisions to invest in foreign real estate. Growing building costs will also result in higher home prices and everyday living expenses due to rising inflation in Thailand or rising oil prices. As a result, in our example study, we can observe residential constructions from Scandinavian developers. ^32^ In addition to tourists from Scandinavia, ^2^^,^^5^ reports a noticeable increase in visitors from Russia, who also have a strong purchasing power; this is encouraging for upcoming tourist and lodging initiatives. ^33^ In more detail, the need for standardized accommodations is brought on by the fact that potential tourists hail from a variety of nations and that many of them demand excellent quality and security due to the length of time they will spend in long-stay lodgings. The government has never established or initiated the initiative on its own, although it now offers conventional guarantee services and some market promotion. ^34^ In some cases, tourism can be a significant factor in revitalizing a rural economy. To alleviate poverty in a remote community in Thailand's northeast, this article suggests implementing a tourism micro cluster model. Using principles gleaned from a comprehensive literature survey, the project also addressed the theoretical underpinnings of a tourism micro cluster model for a rural hamlet in Thailand. ^35^ Developers with qualified operations related to tourism accommodations, such as hotels, retirement homes, care centers, dedicated health facilities, and long-stay businesses, can get investment incentives from responsible sectors like Board of Investment (BOI). ^36^ Throughout this case study of Thailand, the author places special emphasis on the presenting of findings from quantitative and qualitative studies of urban tourism in the cities of Chiang Mai and Phuket. ^37^ Only the private sector creates initiatives and engages in active marketing. Once more, for visitors from wealthy nations with considerable purchasing power, such as Scandinavian nations, the cost of a house is not their major issue; rather, it is the process of buying a property. In this study, we evaluated the theoretical and conceptual frameworks and examined the Thailand tourist industry

## 5. Conclusions

This study promotes the notion of inviting long-stay tourists by outlining significant prospects and stated challenges to offer solutions to these issues. Ultimately, an analysis is meant to produce some helpful recommendations for practitioners and, ideally, future policymakers. Finally, it is necessary to gather data to develop a central database for long-stay accommodations; subsequent studies will then be able to use a more exact quantitative technique. Additional research should concentrate on significant aspects of public policy, such as the length of time a visa is valid, who owns the land, how tourist information is provided, and how tourism affects public relations. It is recommended that some case studies be researched, such as Thailand's neighbors Malaysia and Singapore, not only because these countries are Thailand's rivals but also because they are excellent examples. It is anticipated that the continued popularity of tourism in Thailand will be maintained by a development in this sector, which will in turn promote the economy of the nation.

## Data Availability

All data underlying the results is available as part of the article and no additional source data are required.
