# Supplementary material for: An analysis of the possibilities and challenges of long stay tourism in Thailand
Source: F1000Res. 2023 Nov 6;12:43. Originally published 2023 Jan 11. [Version 2] doi: 10.12688/f1000research.128437.2 (PMC9902795; doi:10.12688/f1000research.128437.2)
Supplement: Peer review history

# Reviewer report: Reviewer response for version 1

**DOI:** 10.5256/f1000research.141028.r159819

**Published:** 2023-02-06

**Authors:** Anila Thomas

**Affiliations:** 1 Department of Tourism & Travel Management, Jyoti Nivas College Autonomous, Bengaluru, India

There is a widespread belief that the expenditure of lodging is an important factor of nearly every vacation; thus, it's essential to think about accommodation expansion when attempting to attract visitors from other countries. Long-stay tourism is important relatively because long visitor stays contribute more revenue generation. The main target are tourists from countries with high living costs, extreme cold climates, and ageing populations. The tourist demographic patterns may expect to change in the coming years, causing the development of new markets, especially, home for retired communities, as an important segment of long-stay tourism.

 The author emphasizes the presentation of findings from quantitative and qualitative studies of urban tourism in the cities of Chiang Mai and Phuket. The current research promotes the idea of encouraging long-stay tourists by identifying vast potential of the region and also indicated the various challenges faced by the service providers and communities in order to provide solutions to the mentioned problems. The sustained prominence of tourism sector in Thailand is expected to be balanced by further expansion of infrastructure and implementation of policy initiatives, that might enhance the country's economic growth.

Is the work clearly and accurately presented and does it cite the current literature?

Yes

If applicable, is the statistical analysis and its interpretation appropriate?

Yes

Are all the source data underlying the results available to ensure full reproducibility?

Yes

Is the study design appropriate and is the work technically sound?

Yes

Are the conclusions drawn adequately supported by the results?

Yes

Are sufficient details of methods and analysis provided to allow replication by others?

Yes

Reviewer Expertise:

Tourism Planning and Development, Community-Based Tourism, Sustainability practices and Tourism Development

I confirm that I have read this submission and believe that I have an appropriate level of expertise to confirm that it is of an acceptable scientific standard.

# Reviewer report: Reviewer response for version 1

**DOI:** 10.5256/f1000research.141028.r159822

**Published:** 2023-02-06

**Authors:** Thanam Subramaniam

**Affiliations:** 1 Centre for Research and Innovation in Tourism (CRiT) & School of Hospitality, Tourism and Events, Taylor’s University, Subang Jaya, Malaysia

It is an interesting topic for the current tourism situation which is in the rejuvenation phase. However, there are a few limitations in the study where the need of the study (problem statement) is unclear.

- Why has the author selected long-stay tourism in Thailand and what is the need for it?
- The methodology of the study can be improved by clearly explaining how the data was obtained. Is this content analysis?
- There is no literature review and underpinning theories in the study.
- Suggested having clear research objectives and answering the objectives through various outcomes.
- Recommended to have constructive arguments in literature review and outcome discussion. 

Is the work clearly and accurately presented and does it cite the current literature?

Partly

If applicable, is the statistical analysis and its interpretation appropriate?

Yes

Are all the source data underlying the results available to ensure full reproducibility?

Yes

Is the study design appropriate and is the work technically sound?

Partly

Are the conclusions drawn adequately supported by the results?

Partly

Are sufficient details of methods and analysis provided to allow replication by others?

Partly

Reviewer Expertise:

Tourism experiences, tourists behaviour, tourism economics, pro-environmental behavior, ecotourism, sustainable tourism, place attachment.

I confirm that I have read this submission and believe that I have an appropriate level of expertise to confirm that it is of an acceptable scientific standard, however I have significant reservations, as outlined above.

# Reviewer report: Reviewer response for version 1

**DOI:** 10.5256/f1000research.141028.r159823

**Published:** 2023-02-06

**Authors:** I Gusti Bagus Rai Utama

**Affiliations:** 1 Dhyana Pura University, Dalung, Indonesia

Abstract

- In the abstract it is necessary to include the method used in this study. and also need to include the conclusions of the results of this study.

Introduction

- The introduction has explained the fact that tourists with long stays are the dream of destination managers. 

Conceptual framework

- Theoretical study is sufficient, it is only necessary to explain the difference between tourists on vacation and immigrants with the reason for traveling with the definition set by the UN-WTO.

Results

- Need to explain why the data analyzed is data from 2010 to 2017? This is to ensure that the data is still feasible for analysis and is still relevant to current issues related to length of stay.

Conclusions

- In the concluding section, it is necessary to explain the contribution of the results of this research to science, and to tourism actors.

Is the work clearly and accurately presented and does it cite the current literature?

Yes

If applicable, is the statistical analysis and its interpretation appropriate?

Yes

Are all the source data underlying the results available to ensure full reproducibility?

Yes

Is the study design appropriate and is the work technically sound?

Yes

Are the conclusions drawn adequately supported by the results?

Yes

Are sufficient details of methods and analysis provided to allow replication by others?

Yes

Reviewer Expertise:

Tourism Management

I confirm that I have read this submission and believe that I have an appropriate level of expertise to confirm that it is of an acceptable scientific standard.
